# Choice of home blood pressure monitoring device: the role of device characteristics among Alaska Native and American Indian peoples

**DOI:** 10.1186/s12872-021-02449-w

**Published:** 2022-01-28

**Authors:** Ashley F. Railey, Denise A. Dillard, Amber Fyfe-Johnson, Michael Todd, Krista Schaefer, Robert Rosenman

**Affiliations:** 1 Department of Sociology, Indiana University, Bloomington, IN USA; 2 Institute for Research and Education to Advance Community Health (IREACH), Elson S. Floyd College of Medicine, Washington State University, Seattle, WA USA; 3 Southcentral Foundation, Anchorage, AK USA

**Keywords:** Blood pressure, Home blood pressure monitoring, Preferences, Alaska Native, American Indian

## Abstract

**Background:**

Home blood pressure monitoring (HBPM) is an effective tool in treatment and long-term management of hypertension. HBPM incorporates more data points to help patients and providers with diagnosis and management. The characteristics of HBPM devices matter to patients, but the relative importance of the characteristics in choosing a device remains unclear.

**Methods:**

We used data from a randomized cross-over pilot study with 100 Alaska Native and American Indian (ANAI) people with hypertension to assess the choice of a wrist or arm HBPM device. We use a random utility framework to evaluate the relationship between stated likely use, perceived accuracy, ease of use, comfort, and participant characteristics with choice of device. Additional analyses examined willingness to change to a more accurate device.

**Results:**

Participants ranked the wrist device higher compared to the arm on a 5-point Likert scale for likely use, ease of use, and comfort (0.3, 0.5, 0.8 percentage points, respectively). Most participants (66%) choose the wrist device. Likely use (wrist and arm devices) was related to the probability of choosing the wrist (0.7 and − 1.4 percentage points, respectively). Independent of characteristics, 75% of participants would be willing to use the more accurate device. Ease of use (wrist device) and comfort (arm device) were associated with the probability of changing to a more accurate device (− 1.1 and 0.5 percentage points, respectively).

**Conclusion:**

Usability, including comfort, ease, and likely use, appeared to discount the relative importance of perceived accuracy in the device choice. Our results contribute evidence that ANAI populations value accurate HBPM, but that the devices should also be easy to use and comfortable to facilitate long-term management.

**Supplementary Information:**

The online version contains supplementary material available at 10.1186/s12872-021-02449-w.

## Background

Home blood pressure monitoring (HBPM) is an effective tool in the treatment and long-term management of hypertension [1]. Incorporating regular monitoring of blood pressure at home into treatment plans may help improve hypertension control by increasing the number of readings, reducing white-coat and masked hypertension, facilitating patient understanding of blood pressure, and detecting variability in blood pressure [2]. The primary devices for home monitoring include a wrist or an arm cuff, with the arm cuff device being more accurate [3–5]. Long-term use of HBPM devices involves the acceptance of the device [6–8] and device characteristics such as usability and perceptions of accuracy [9–11]. An uncomfortable device with uncertain accuracy evokes negative attitudes towards HBPM [10, 12] but the ease of use enhances the overall experience and may overcome limitations due to comfort [10, 11]. While these device characteristics are known to matter to patients, what is not known is the relative importance of each of these characteristics in the patient’s device choice.

Understanding device choice may help improve long-term use of HBPM, which is important for populations at-risk for hypertension. Among Alaska Native and American Indian people (ANAI), the prevalence of high blood pressure and hypertension has increased since the early 1990s [13]. Recent estimates place the prevalence between 25 and 40%, though the prevalence may be higher due to disparities in undiagnosed hypertension [14, 15]. At the same time, ANAI communities in Alaska face considerable barriers to treating hypertension, including scarcity of nearby clinics, overlapping comorbidities, and historical mistrust of health services [16, 17]. HBPM may be an important tool for decreasing these barriers to treating hypertension if used long-term. Knowing how patients choose a HBPM device will help providers understand how to assist with the choice and overall hypertension management.

Assessments of patient preferences (for reviews see [18, 19]) about care [20, 21] often rely on a random utility framework to quantify differences in patient demand. The framework is ideal for evaluating patient preferences for care by capturing tradeoffs between technical characteristics such as complications [22] and wait times [23, 24], non-technical characteristics such as interpersonal interactions with staff [25] and the physical environment [23], and evolving expectations [26]. Qualitative accounts of preferences for care find that both technical characteristics and non-technical characteristics influence preferences through perceptions of quality. Extending the random utility framework to HBPM device choice optimizes on the growing market for devices as a commodity, the role of patient preferences in guiding future device innovation [27], and as a hypertension management strategy in clinical practice [28–30].

In this paper, we assessed the relationship between HBPM device choice and individual preferences for device characteristics among a study population of ANAI people with self-reported hypertension at Southcentral Foundation, a nonprofit, tribally owned and operated health care center in Southcentral Alaska. The provision of either an arm or a wrist HBPM device, coupled with limited patient experience with HBPM devices at Southcentral Foundation, prompted us to use a random utility framework to evaluate tradeoffs between the two devices [26, 31]. The findings from our analysis directly inform the provision of either an arm or wrist HBPM device at Southcentral and suggest potential barriers to long-term use.

## Methods

### Setting and study sample

The data used for this analysis come from a randomized cross-over pilot study at Southcentral Foundation (SCF). SCF provides primary care services to over 65,000 ANAIs living in Southcentral Alaska, including Anchorage, the rural Matanuska Susitna Borough, and 55 remote villages [32]. SCF services are “prepaid” based on legislative agreements between the United States and tribes.

**Fig. 1:**
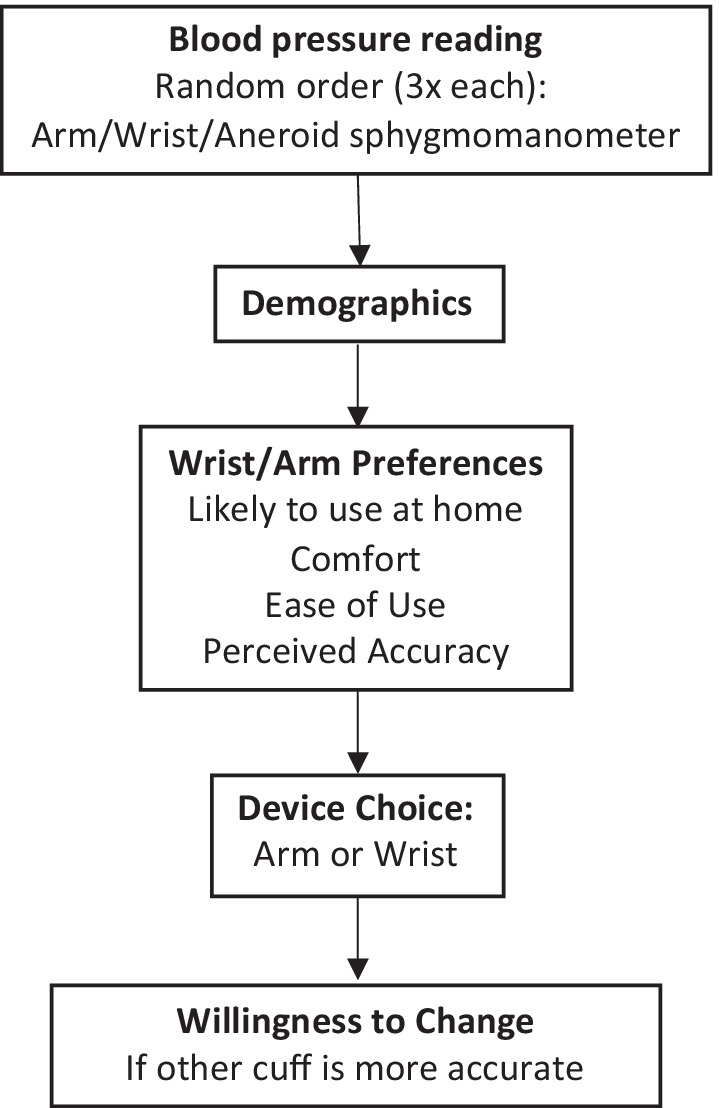
Order of events in study

SCF conducted a 2-week cross-over study to evaluate the preferences and performance of a wrist (Omron Series 7, BP654) and an arm (Omron Series 10, BP786N) HBPM device in a sample of 100 ANAI adults with hypertension. At baseline, research staff measured arm and wrist circumference. Participants then had their blood pressure measured with both HBPM devices and from a calibrated aneroid sphygmomanometer. The order of devices was randomized across participants and device readings were not blinded. Following the blood pressure measurements, participants received a questionnaire containing information on basic demographics and responses to the arm and wrist cuff devices, including likely use at home, perceived device accuracy, ease of use, and comfort. Participants finished by stating their choice for either the arm or the wrist device to use at home and whether they would change to the other device if it was found to be more accurate (see Fig. 1 for data collection order). For this pilot study, participants then took each device home for a 1-week trial, with the order randomized across participants. This study was approved by the Alaska Area Institutional Review Board and tribal leadership of Southcentral Foundation and the Alaska Native Tribal Health Consortium.

### Measures

The device choice was between a wrist or arm cuff. The characteristics assessed included likely use at home, perceived accuracy, ease of use, and comfort ranked on a 5-point Likert scale. We retained the full Likert scale responses specified as a linear relationship to maintain degrees of freedom, as well as to model the decision between cuff devices on a continuum. Ease of use and comfort were assessed given their frequent citation as the distinguishing features of blood pressure monitoring devices [10, 11]. Perceived accuracy was elicited as a measure of perceived quality after the participants had their blood pressure taken on the three devices [33–35]. The stated likelihood of use reflects the participants’ perceived self-motivation to routinely use the chosen device to measure blood pressure over an extended amount of time [36, 37]. After choosing the preferred device, the participants were asked their willingness to change to the other device if the other proved more accurate. The question intended to evaluate the stability of preferences in the presence of additional information. We assess the choice to change as a binary outcome between those who were ‘very willing’ to change devices from those who were ‘willing, but not happy about it’ and ‘not willing and would want one I chose anyway’. This separates strong preferences (‘very willing’) from other, potentially malleable preferences (‘unwilling/willing but hesitant’). In all analyses, we controlled for age as a continuous variable, gender (woman/men), whether a participant has any college education as a dichotomous variable, annual household income across three categories (< $35,000; $35–59,999; $60,000+), and device fit (arm and wrist circumference).

### Statistical analysis

We employed summary statistics and logistic regressions to evaluate the relationships between each device’s characteristics, likelihood of using each device, participant demographics, and the choice of device. Following the ordering of questions and random utility framework, we separately evaluated the choice of the wrist device and the willingness to change devices if the other cuff device was more accurate [38]. Non-response on any of the variables was treated as missing values and excluded from the analysis (n = 19) after assessing for non-randomness. Less than 10% of any one variable exhibited missing values. All analyses were performed in Stata 16.

## Results

Table 1 outlines the summary statistics for the participant demographics and select device characteristics. The average participant age was 51 years old and 60% were women. Most of the participants reported some college/college education (64%) compared to less than college. Fewer than 10% had upper arm or wrist circumferences that exceeded the manufacturer’s recommended size (≤ 43.2 cm and ≤ 21.5 cm, respectively). The wrist device on average read higher than the arm and with greater variation (for full details on device performance see [39]). Of the two devices, 66% initially chose the wrist and 34% chose the arm. When asked if the participant was willing to take home the other, non-preferred device if it was more accurate, 75% were very willing to change and 25% were unwilling or willing but hesitant.

**Table 1:** Selected characteristics of HBPM device preference study participants, n = 100

Variable	n = 100
Age, mean (SD)	51 (12)
Gender	
Men	40
Income	
$0–34,999	44
$35–59,999	32
$60,000+	24
Education	
Some college/college	64
Device choice	
Wrist	66
Willingness to change	
Yes	75
Wrist circumference in cm, mean (SD)	18 (2)
Arm circumference in cm, mean (SD)	35 (6)
Sphygmomanometer blood pressure in mmHg	133/80 (14/11)
Wrist cuff device blood pressure in mmHg, mean systolic/diastolic (SD)	139/85 (20/15)
Arm cuff device blood pressure in mmHg, mean systolic/diastolic (SD)	131/84 (17/12)

Responses from baseline survey at Southcentral Foundation

*SD* standard deviation

Table 2 shows the ranking of device characteristics on a continuous scale. The full distribution of the rankings appears in Additional file 1: Table S1. Overall, participants ranked the wrist device higher compared to the arm on likelihood of use (2.8 vs. 2.5), ease of use (3.6 vs. 3.1), and comfort (3.6 vs. 2.8). The participants ranked the arm device higher for perceived accuracy (2.7 vs. 2.4). These trends remain when comparing the difference in device rankings and by choice of device (Additional file 1: Table S2).

**Table 2:** Average rank of device characteristic, stratified by device

	Device		
	Wrist	Arm	*P* value
Overall rank (scale 1–5)			
Likelihood of use (n = 98)	2.8	2.5	0.05
Perceived accuracy (n = 91)	2.4	2.7	< 0.01
Ease of use (n = 96)	3.6	3.1	< 0.01
Comfort (n = 96)	3.6	2.8	< 0.01

Participants provided responses for both devices. Responses from baseline survey at Southcentral Foundation. Two-sided *t* tests. Rankings based on a 5-point Likert scale, where 1 = “not at all likely”, “completely inaccurate”, or “very dissatisfied” and 5 = “extremely likely”, “completely accurate,” or “very satisfied”

The results on participant choice of the wrist cuff appear in Table 3. The likelihood of using the wrist and arm devices were associated with choosing the wrist device (0.7 percentage point and − 1.4 percentage points, respectively). For example, ‘not at all likely’ to use the wrist device was associated with a 0.4 probability of choosing the wrist and ‘extremely likely’ was associated with a 0.9 probability of choosing the wrist device. Similarly, ‘not at all likely’ to use the arm device was related to a 0.9 probability of choosing the wrist device while ‘extremely likely’ to use the arm device was related to a 0.2 probability of choosing the wrist device. Income was marginally associated with choice of wrist device. Additional specifications supporting the strength of relationship between likelihood of use, the device characteristics, and probability of choosing the wrist device appear in the supplementary materials (Additional file 1: Table S3 and S4).

**Table 3:** Device and participant characteristics associated with choosing the wrist cuff device among HBPM study participants (n = 81)

Characteristic	Marginal effects^a^	[95% conf. interval]
Wrist ranking		
Likelihood of use	0.7	[0.2 1.2]
Perceived accuracy	0.6	[− 0.2 1.5]
Ease of use	− 0.1	[− 1.4 1.3]
Comfort	0.6	[− 0.6 1.9]
Arm ranking		
Likelihood of use	− 1.4	[− 2.5 − 0.4]
Perceived accuracy	− 0.1	[− 1.1 0.9]
Ease of use	− 0.8	[− 2.4 0.9]
Comfort	0.3	[− 0.6 1.1]
Age	0.1	[− 0.9 1.2]
Education		
Some college/college	0.0	[− 0.2 0.2]
Income		
35–59,999	− 0.0	[− 0.2 0.2]
60,000+	− 0.2	[− 0.4 0.0]
Gender		
Men	− 0.1	[− 0.3 0.1]
Circumference		
Wrist	1.9	[− 2.2 6.0]
Mid-upper arm	0.0	[− 3.1 3.1]

Responses from baseline survey at Southcentral Foundation. Binary outcome logit model where wrist device = 1 and arm device = 0. Estimated with robust standard errors

^a^Marginal effects are interpreted for continuous regressors as elasticities at the mean where the dependent, outcome variables and independent variables change at a constant rate. The categorical variables are the marginal values taken as an approximate percentage effect of the variable in response to a discrete change from zero to one, while holding all other parameters constant

Table 4 presents the results on the characteristics associated with the willingness to change to the other device if it was found to be more accurate. The ease of using the wrist device and the comfort of the arm device were associated with the probability of changing devices. Being ‘very dissatisfied’ with the ease of use and comfort was associated with a 0.9 and 0.5 probability, respectively, of being willing to change devices. Being ‘very satisfied’ with the ease of using the wrist device and the comfort of the arm device was associated with a 0.5 and 0.9 probability, respectively, of being willing to change devices. The comfort of the arm device had the largest association with the probability of changing devices among the arm characteristics (0.5 percentage point). The likelihood of using either device appeared to be minimally associated with the willingness to change devices. Age was associated with the willingness to change to the more accurate device (− 0.9 percentage point).

**Table 4:** Device and participant characteristics associated with willingness to change to a more accurate device among HBPM study participants (n = 81)

	Marginal effects^a^	[95% conf. interval]
Wrist ranking		
Likelihood of use	0.2	[− 0.2 0.6]
Perceived accuracy	0.1	[− 0.4 0.5]
Ease of use	− 1.1	[− 2.1 0.0]
Comfort	0.7	[− 0.2 1.5]
Arm ranking		
Likelihood of use	0.1	[− 0.4 0.5]
Perceived accuracy	0.2	[− 0.4 0.8]
Ease of use	− 0.5	[− 1.2 0.3]
Comfort	0.5	[0.1 1.5]
Choice of wrist cuff	0.2	[− 0.1 0.5]
Age	− 0.9	[− 1.7 0.0]
Education		
Some college/college	0.0	[− 0.2 0.2]
Income		
35–59,999	0.0	[− 0.2 0.2]
60,000+	− 0.1	[− 0.3 0.2]
Gender		
Men	0.0	[− 0.2 0.2]
Circumference		
Wrist	− 0.5	[− 3.3 2.2]
Mid-upper arm	− 0.5	[− 2.7 1.7]

Responses from baseline survey at Southcentral Foundation. Binary outcome logit model where willingness to change = 1 and ‘unwilling/willing but hesitant’ = 0. Estimated with robust standard errors

^a^Marginal effects are interpreted for continuous regressors as elasticities at the mean where the dependent, outcome variables and independent variables change at a constant rate. The categorical variables are the marginal values taken as an approximate percentage effect of the variable in response to a discrete change from zero to one, while holding all other parameters constant. More accurate defined as the opposite of the chosen device. For example, for those who chose the wrist device, the more accurate device was presented as the arm

## Discussion

Our study evaluated the role of preferences for HBPM device characteristics in the choice of either a wrist or an arm cuff device. We found that the likelihood of use at home was strongly associated with choice of device. Likelihood of use may reflect perceptions of future self (i.e., self-efficacy, motivation, self-control, executive function) [40], which would lend support to extant studies that cite the burden of taking blood pressure readings over an extended amount of time as a determinant of use [41]. Age and income may likewise be capturing self-management constraints through a potential relationship with the portability of the arm cuff [10]. Our results accord with the literature on blood pressure management decisions [42] and long-term use [26, 43] to suggest that patient constraints will likely influence choosing the most accurate device and willingness to change devices despite the substantial reductions in structural barriers from shifting to home monitoring.

Device usability has been cited as a significant barrier to choosing the more accurate arm cuff [10]. Our study further suggests that the tangible measures of usability (ease of use and comfort) may influence long-term use through the willingness to change cuffs [41]. Similar to comparisons between ambulatory and home blood pressure monitoring [12, 44], the more comfortable and easier to use wrist device was preferred to the arm device despite lower accuracy. A clear tradeoff in the decision to change to the more accurate device occurred between not wanting to change from the ease of using the wrist device to the (dis)comfort of the arm device. This occurs regardless of device cuff fit based on arm and wrist circumference. With respect to facilitating long-term use, increasing the comfort of the arm device jointly with increased information on the accuracy of the arm device may help reduce the relative importance of easy use in the HBPM decision.

Participants appeared to discount their perceptions of accuracy in the choice of device. Perceived accuracy was not the strongest or most consistent factor to influence choice of device. Accuracy and reliability reflect the essential metrics of quality from a clinical perspective [1] but user perceptions of quality may vary from clinical standards due to instances of inaccurate or unreliable HBPM blood pressure readings. This occurs in the absence of opportunities to learn about product quality [45–47], or in the case of the choice between an arm or a wrist cuff device, when the choices are substitutes to collect clinical data. Over time, a higher than expected reading may lead to increased patient use of the HBPM device either to continually reassess the accuracy of the reading [48] or because the patient believes the reading is true and sees a need for continual monitoring [33, 49]. Conversely, patients have been shown to prefer devices that report lower than expected readings [34], which would have the opposite effect on long-term use. Thus, the relationship between perceived device accuracy and adherence over time warrants further investigation.

Relationships with providers influence perceptions of quality and can be especially important in traditionally disadvantaged and at-risk populations [50]. In the case of choosing between an arm or a wrist device, information on the actual device accuracy was limited during the baseline visit, such that participants would have to infer the quality of the two devices based on previous experiences with high quality care at SCF. Patients at SCF can expect exceptional access and availability to primary healthcare services [32], but both the arm and the wrist were new devices which may have signaled uniform quality, especially in the absence of price. The ambiguous effect of perceived device accuracy on long-term outcomes offers the potential for long-term feedback about HBPM from providers or treatment modifications to adjust for HBPM reading trends [51]. The negative impact of choosing a less accurate device on clinical outcomes may be minimal when coupled with the standard of patient care that is found at SCF.

## Strengths and limitations

Our study benefited from occurring alongside a clinical trial in which participants use a HBPM device in a healthcare center that intends to offer HBPM to its patients. The consequentiality of their responses provided a strong incentive to reveal true preferences [52]. Beyond the trial setting, the choice between an arm or a wrist device appropriately reflects the current decision environment for HBPM devices [53]. Our subsequent ability to assess device choice following a random utility framework gave us the advantage of defining the importance of device characteristics and demographics in the decision.

The primary limitation of using pilot data for secondary, exploratory analyses is sample size. This included confining our ability to explore how ranking perceptions of accuracy and actual accuracy interact to influence the choice of device or using traditional mixed, latent class models in the random utility framework to retrieve clinically meaningful changes in device characteristics [54]. Omitted variable bias presents the greatest threat to our identification strategy by not capturing additional variables related to the participant, device portability, [44] or previous experience [55]. Finally, while trial protocols attempted to reduce social desirability bias in responses and researchers’ influence on perceptions of accuracy, we cannot know the extent of variation in conversations between researchers and participants, including whether participants saw baseline blood pressure measurements.

## Conclusions

The results from this study help demonstrate to providers that ANAI populations recognize the need for accurate blood pressure monitoring, but device usability cannot be sacrificed. Particularly considering the patient burden of repeated measurements per day, over multiple months, or years, underestimating the prominence of device usability is problematic. Improving the comfort of the arm device to reduce pinching or ensuring the correct device size may address initial hesitations toward the device. Devising plans between the patient and provider to alleviate the burden of use over time is an initial approach in the absence of device improvements. Importantly, relationships with providers influence perceptions of quality and can be leveraged to emphasize the subtleties in accuracy and reliability, which may impact treatment outcomes. This holds true in our ANAI sample and provides encouragement for broader acceptance of HBPM among people in traditionally underserved locations.

## Supplementary Information

**Additional file 1.** Additional summary statistics and alternative model specifications.

## Data Availability

The datasets generated during and/or analyzed during the current study are not publicly available due to tribal sovereignty over research data.

## Abbreviations

ANAI: Alaska Native and American Indian
HBPM: Home blood pressure monitoring
SCF: Southcentral foundation
