# Supplementary material for: Choice of home blood pressure monitoring device: the role of device characteristics among Alaska Native and American Indian peoples
Source: BMC Cardiovasc Disord. 2022 Jan 28;22:19. doi: 10.1186/s12872-021-02449-w (PMC8796453; doi:10.1186/s12872-021-02449-w)
Supplement: Supplementary file 1 — Additional file 1. Additional summary statistics and alternative model specifications. [file 12872_2021_2449_MOESM1_ESM.docx]

**Supplementary Material**

**Choice of home blood pressure monitoring device: the role of device characteristics among Alaska Native and American Indian peoples**

**Authors:**

Ashley F. Railey^1,2+^, Denise A. Dillard^3^, Amber Fyfe-Johnson^2^, Michael Todd^3^, Krista Schaefer^3^, Robert Rosenman^2^

**Author Primary Affiliations:**

1—Department of Sociology, Indiana University

2—Institute for Research and Education to Advance Community Health (IREACH), Elson S. Floyd College of Medicine, Washington State University

3—Southcentral Foundation, Alaska

^+^Work completed during time at Institute for Research and Education to Advance Community Health (IREACH), Elson S. Floyd College of Medicine, Washington State University

**Corresponding Author:**

Ashley F. Railey

Indiana University

1022 E. Third St

Bloomington, IN 47405-7103

[arailey@iu.edu](mailto:arailey@iu.edu)

| **Table S1** Full ranking of device characteristics on a 5-point Likert scale at baseline by device | | |
| --- | --- | --- |
| Variable | Frequency (#) | |
|  | Wrist Device | Arm Device |
| **Device Choice** | 66 | 34 |
| **Likelihood of Use** |  |  |
| Not at all Likely | 7 | 5 |
| Slightly Likely | 4 | 9 |
| Moderately Likely | 16 | 26 |
| Very Likely | 39 | 42 |
| Extremely Likely | 33 | 17 |
| **Perceived Accuracy** |  |  |
| Completely Inaccurate | 5 | 1 |
| Very Inaccurate | 8 | 2 |
| Neutral | 42 | 33 |
| Very Accurate | 30 | 42 |
| Completely Accurate | 9 | 16 |
| **Ease of Use** |  |  |
| Very Dissatisfied | 1 | 3 |
| Slightly Dissatisfied | 1 | 4 |
| Neutral | 6 | 12 |
| Slightly Satisfied | 13 | 36 |
| Very Satisfied | 76 | 44 |
| **Comfort** |  |  |
| Very Dissatisfied | 1 | 8 |
| Slightly Dissatisfied | 2 | 6 |
| Neutral | 5 | 18 |
| Slightly Satisfied | 17 | 27 |
| Very Satisfied | 73 | 39 |
| **Willingness to Change** |  |  |
| Unwilling/Willing but hesitant | 13 | 12 |
| Willing | 53 | 22 |
| Notes: Participants ranked both the arm and the wrist devices. Frequencies may not add up to 100 across variables due to missing values. | | |

| **Table S2** Device characteristic rankings stratified by device choice | | | | |
| --- | --- | --- | --- | --- |
|  |  | Choice of device | | |
|  | Overall | Wrist | Arm | P value |
| Wrist |  |  |  |  |
| Accuracy | 2.35 | 2.67 | 1.76 | < 0.001 |
| Ease of use | 3.58 | 3.73 | 3.48 | 0.05 |
| Comfort | 3.64 | 3.71 | 3.35 | 0.01 |
| Likelihood of use | 2.77 | 3.17 | 2.03 | < 0.001 |
| Arm |  |  |  |  |
| Accuracy | 2.72 | 2.73 | 2.69 | 0.76 |
| Ease of use | 3.14 | 3.00 | 3.38 | 0.02 |
| Comfort | 2.78 | 2.58 | 3.14 | 0.01 |
| Likelihood of use | 2.51 | 2.19 | 3.07 | < 0.001 |
| Difference (Arm-Wrist) |  |  |  |  |
| Accuracy | 0.40 | 0.12 | 0.91 | 0.01 |
| Ease of use | 0.51 | -0.70 | -0.13 | 0.03 |
| Comfort | -0.79 | -1.11 | -0.16 | < 0.01 |
| Likelihood of use | -0.32 | -0.94 | 0.97 | < 0.001 |
| Notes: Two-sided t-tests. Rankings based on a 5-point Likert scale, where 1= “not at all likely”, “completely inaccurate”, or “very dissatisfied” and 5=” extremely likely”, “completely accurate,” or “very satisfied”. Participants provided responses for both devices. Responses from baseline survey at Southcentral Foundation. | | | | |

| **Table S3** Correlations between device characteristics and devices | | | | | | | | | |
| --- | --- | --- | --- | --- | --- | --- | --- | --- | --- |
|  | **Arm** | | | |  | **Wrist** | | | |
|  | Likelihood of Use | Perceived Accuracy | Ease of Use | Comfort |  | Likelihood of Use | Perceived Accuracy | Ease of Use | Comfort |
| **Arm** |  |  |  |  |  |  |  |  |  |
| Likelihood of Use | 1 |  |  |  |  |  |  |  |  |
| Perceived Accuracy | 0.37* | 1 |  |  |  |  |  |  |  |
| Ease of Use | 0.53*** | 0.42*** | 1 |  |  |  |  |  |  |
| Comfort | 0.65*** | 0.38*** | 0.68* | 1 |  |  |  |  |  |
| **Wrist** |  |  |  |  |  |  |  |  |  |
| Likelihood of Use | -0.01 | 0.18* | -0.001 | -0.09 |  | 1 |  |  |  |
| Perceived Accuracy | -0.23*** | 0.16* | -0.06 | -0.17* |  | 0.41* | 1 |  |  |
| Ease of Use | -0.04 | 0.23 | 0.07 | 0.01 |  | 0.23*** | 0.20** | 1 |  |
| Comfort | -0.03 | 0.04 | 0.12 | 0.03 |  | 0.23*** | 0.12 | 0.64*** | 1 |
| Notes: Spearman's order correlations between devices. Statistical significance: *** p < 0.01, ** p < 0.05, * p < 0.10 | | | | | | | | | |

| **Table S4** Associations of the difference in device rankings on choice of wrist device and willingness to change devices | | | | | | | | | |
| --- | --- | --- | --- | --- | --- | --- | --- | --- | --- |
|  | Choice of Wrist Device | | | |  | Willingness to Change Devices | | | |
|  | Marginal Effects^a^ | P value | [95% Conf. | Interval] |  | Marginal Effects^a^ | P value | [95% Conf. | Interval] |
| Arm-Wrist Ranking |  |  |  |  |  |  |  |  |  |
| Likelihood of Use | -0.67 | 0.04 | -1.30 | -0.04 |  | 0.00 | 0.58 | -0.01 | 0.02 |
| Perceived accuracy | -0.31 | 0.16 | -0.74 | 0.12 |  | 0.02 | 0.29 | -0.01 | 0.05 |
| Ease of use | 0.01 | 0.24 | -0.01 | 0.03 |  | -0.02 | 0.74 | -0.15 | 0.11 |
| Comfort | -0.01 | 0.81 | -0.06 | 0.04 |  | -0.11 | 0.35 | -0.33 | 0.12 |
| Choice of Cuff (Wrist) |  |  |  |  |  | 0.25 | 0.05 | 0.00 | 0.49 |
| Age | 0.02 | 0.98 | -1.00 | 1.03 |  | -0.49 | 0.14 | -1.14 | 0.16 |
| Education |  |  |  |  |  |  |  |  |  |
| Some college/college | 0.00 | 0.97 | -0.17 | 0.16 |  | -0.01 | 0.96 | -0.22 | 0.21 |
| Income |  |  |  |  |  |  |  |  |  |
| 35-59,999 | -0.03 | 0.76 | -0.22 | 0.16 |  | -0.02 | 0.86 | -0.24 | 0.20 |
| 60,000+ | -0.23 | 0.02 | -0.43 | -0.03 |  | -0.03 | 0.79 | -0.28 | 0.21 |
| Gender |  |  |  |  |  |  |  |  |  |
| Men | -0.06 | 0.46 | -0.23 | 0.10 |  | 0.05 | 0.64 | -0.15 | 0.24 |
| Observations | 81 | | | |  | 81 | | | |
| Notes: Binary outcome logit model. ^a^Marginal effects are interpreted for continuous regressors as elasticities at the mean where the dependent, outcome variables and independent variables change at a constant rate. The categorical variables are the marginal values taken as an approximate percentage effect of the variable in response to a discrete change from zero to one, while holding all other parameters constant. P value estimated with two-tail tests of significance and robust standard errors. Characteristics reported at baseline survey for Southcentral Foundation participants. Arm-Wrist Ranking measured as the difference in the arm ranking minus the wrist ranking. More accurate defined as the opposite of the chosen device. For example, for those who chose the wrist device, the more accurate device was presented as the arm. | | | | | | | | | |
